# Bevacizumab as Rescue Therapy for GI Bleeding in Hereditary Hemorrhagic Telangiectasia

**DOI:** 10.7759/cureus.106279

**Published:** 2026-04-01

**Authors:** Abay A Gobezie, Sneha Adidam, Mekdem Bisrat, Samrawit W Zinabu, Wondwossen Alemayehu, Kalkidan S Alayu, Naga Sai Shravan Turaga, Farshad Aduli, Angesom Kibreab, Miriam B Michael

**Affiliations:** 1 Internal Medicine, Howard University Hospital, Washington, DC, USA; 2 Public Health, Project HOPE, Washington, DC, USA; 3 Gastroenterology, AdventistHealth System, Altamonte Springs, USA; 4 Gastroenterology, Howard University Hospital, Howard University College of Medicine, Washington, DC, USA; 5 Gastroenterology, Howard University, Washington, DC, USA; 6 Internal Medicine, Howard University, Washington, DC, USA; 7 Internal Medicine, University of Maryland, Baltimore, USA

**Keywords:** angiodysplasia, anti-angiogenic therapy, arteriovenous malformation, bevacizumab, gastrointestinal bleeding, hereditary hemorrhagic telangiectasia, vegf

## Abstract

Hereditary hemorrhagic telangiectasia (HHT) is an autosomal dominant vascular disorder characterized by abnormal blood vessel formation, frequently involving the gastrointestinal (GI) tract. GI involvement can result in diffuse mucosal telangiectasis, chronic bleeding, iron-deficiency anemia, and transfusion dependence. Conventional treatments often provide limited benefit in patients with widespread disease. Bevacizumab, a monoclonal antibody targeting vascular endothelial growth factor (VEGF), has emerged as a potential disease-modifying agent through its anti-angiogenic properties.

We report a case of a male patient with genetically confirmed HHT and comorbid pulmonary arterial hypertension who presented with recurrent GI bleeding. The patient had a long-standing history of GI bleeding requiring multiple endoscopic interventions, with findings of arteriovenous malformations throughout the GI tract. Prior management with conventional therapies provided only a transient benefit. The patient subsequently received intravenous bevacizumab induction therapy. Bevacizumab therapy resulted in marked clinical improvement, including hemoglobin stabilization, reduction in overt bleeding, and decreased transfusion requirements. Recurrence of bleeding following therapy discontinuation further supports a direct therapeutic effect. This case contributes to the growing body of evidence that VEGF inhibition is a biologically rational and clinically effective salvage strategy for refractory GI bleeding in HHT. Prospective studies are needed to define optimal dosing, maintenance strategies, and long-term outcomes.

## Introduction

Hereditary hemorrhagic telangiectasia (HHT) results from pathogenic variants in genes encoding components of the transforming growth factor-β (TGF-β) signaling pathway, predominantly endoglin (ENG) and activin receptor-like kinase 1 (ACVRL1), leading to widespread arteriovenous malformations across multiple vascular beds [1,2]. Gastrointestinal involvement affects approximately 25-30% of patients, typically manifesting in the fifth or sixth decade with chronic bleeding from diffuse mucosal telangiectases [3,4]. Diffuse telangiectasia, such as that seen in radiation proctopathy or hereditary hemorrhagic telangiectasia, presents a significant therapeutic challenge when lesions are too widespread or numerous for targeted endoscopic coagulation to be practical or effective. In these cases, systemic approaches, including antifibrinolytic agents, hormonal therapy, or thalidomide, alongside interventional radiology or surgical resection, may be considered when bleeding remains refractory and clinically significant. The resulting iron-deficiency anemia often necessitates repeated transfusions and substantially impairs quality of life, with a subset of patients demonstrating refractory disease despite multimodal conventional therapy including endoscopic ablation, antifibrinolytics, and somatostatin analogs [5,6].

The pathophysiology of vascular malformations in HHT centers on dysregulated angiogenesis secondary to aberrant TGF-β/bone morphogenetic protein (BMP) signaling. Haploinsufficiency of ENG or ACVRL1 disrupts endothelial cell differentiation and vascular remodeling, resulting in structurally abnormal vessels with direct arteriovenous shunting. This mechanistic framework has prompted investigation of anti-angiogenic therapeutics, particularly vascular endothelial growth factor (VEGF) inhibition. Bevacizumab, a humanized monoclonal antibody against VEGF-A, has demonstrated clinically significant reductions in transfusion requirements and improved hemoglobin stability in a small series of HHT patients with refractory bleeding through dual mechanisms as follows: suppression of pathological neovascularization and normalization of dysmorphic vasculature [7,8].

Despite these encouraging preliminary results, bevacizumab remains off-label for HHT-related gastrointestinal bleeding, and robust prospective data are lacking. The anti-angiogenic properties of VEGF inhibition provide compelling biological rationale not only for HHT-associated bleeding but potentially for other etiologies of gastrointestinal angiodysplasia. Given the significant morbidity of refractory GI bleeding in HHT and the limitations of endoscopic approaches in managing diffuse mucosal disease, a systematic investigation of bevacizumab's efficacy, optimal dosing regimens, and response durability is warranted. We present a case of genetically confirmed HHT with medically and endoscopically refractory gastrointestinal bleeding who achieved sustained clinical remission following bevacizumab induction therapy, and discuss the broader implications of anti-VEGF therapy for managing gastrointestinal bleeding.

This work was previously presented as an abstract at the American College of Gastroenterology (ACG) Annual Scientific Meeting in October 2024.

## Case presentation

A 60-year-old male with a medical history of pulmonary arterial hypertension presented to the emergency room with intermittent melena for one month. He reported a history of recurrent gastrointestinal bleeding (GIB) due to arteriovenous malformation (AVM) in the GI tract and was recently diagnosed with hereditary hemorrhagic telangiectasia (HHT).

**Figure 1:**
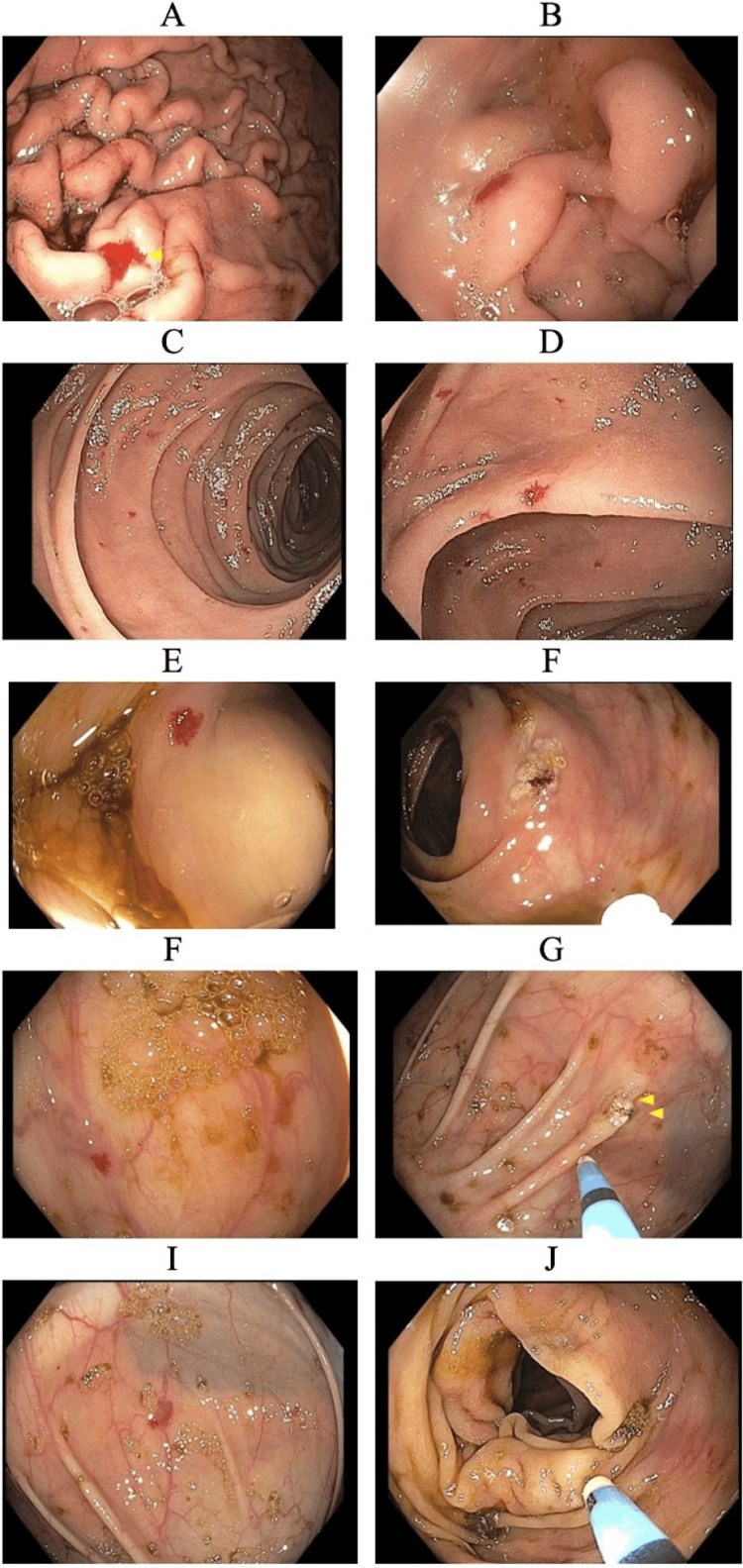
Endoscopic views showing multiple gastrointestinal AVMs across the stomach, duodenum, and colon, with APC treatment. Esophagogastroduodenoscopy showing AVM in the gastric body (A), duodenal bulb (B), second part of the duodenum (C), and third part of the duodenum (D). Colonoscopy showing AVM and bleeding controlled with APC in the ascending colon (E, F), transverse colon (G, H), and hepatic flexure (I, J). APC: argon plasma coagulation; AVM: arteriovenous malformation

Since 2015, the patient has experienced repeated episodes of chronic lower GIB, necessitating multiple blood transfusions and iron infusions. From 2015 to 2023, he underwent upper endoscopy and colonoscopy seven times and video capsule endoscopy once to investigate and control the source of bleeding. Endoscopic findings revealed multiple small (2-4 mm) AVMs in the esophagus, gastric antrum, body, duodenum, and colon. The endoscopic findings are shown in Figures 1A-1J. Bleeding was managed with argon plasma coagulation (APC) via endoscopy and medical management with octreotide and tranexamic acid.

The patient met two out of four consensus diagnostic criteria for HHT, including endoscopic findings and the presence of telangiectasia in the oral cavity. The definitive diagnosis was confirmed through genetic testing. Hematology recommended treatment with bevacizumab infusions. The patient received an initial induction course of eight cycles of biweekly Avastin infusion, which led to an improvement in symptoms and control of bleeding, strongly suggesting a clinical benefit of bevacizumab for HHT patients. However, due to insurance issues, the patient was unable to receive maintenance doses. Consequently, the patient began experiencing lower GIB again.

## Discussion

mechanism of action makes itThis case illustrates the potential role of systemic anti-angiogenic therapy as a disease-modifying treatment for refractory gastrointestinal bleeding. Following eight cycles of biweekly bevacizumab induction therapy, our patient experienced marked clinical improvement, including stabilization of hemoglobin levels, reduction in overt bleeding episodes, and decreased transfusion requirements. The recurrence of bleeding after interruption of therapy further supports a therapeutic effect rather than spontaneous disease fluctuation [9,10].

Hereditary hemorrhagic telangiectasia is fundamentally a disorder of dysregulated angiogenesis driven by impaired TGF-β/BMP signaling and subsequent overactivation of VEGF pathways [11,12]. The resulting fragile, ectatic vasculature predisposes patients to chronic mucosal bleeding. Traditional management strategies, such as argon plasma coagulation, antifibrinolytics, and somatostatin analogs, primarily target the consequences of abnormal vessels rather than the underlying angiogenic drive. In contrast, bevacizumab directly inhibits VEGF-A, thereby suppressing pathological neovascularization and promoting vascular stabilization [7,8].

Given this mechanistic rationale, VEGF inhibition represents a biologically targeted approach to bleeding driven by vascular malformations. Bevacizumab is widely utilized in hematologic and oncologic conditions for its anti-angiogenic properties and has an established safety profile when appropriately monitored. Its systemic mechanism of action makes it particularly appealing in patients with diffuse gastrointestinal telangiectasis, where focal endoscopic therapies are often insufficient [9].

Importantly, the implications of this therapeutic strategy may extend beyond HHT. Angiodysplasia-related gastrointestinal bleeding in non-HHT populations is also characterized by aberrant angiogenesis and increased VEGF expression [13]. Therefore, anti-VEGF therapy could represent a novel treatment paradigm for selected patients with refractory vascular GI bleeding who remain transfusion-dependent despite optimized endoscopic management.

However, several critical questions remain unanswered. Optimal induction and maintenance dosing strategies, duration of therapy, long-term durability of response, and risk-benefit considerations require systematic evaluation. Prospective controlled studies are needed to determine whether bevacizumab should be incorporated earlier in the treatment algorithm for severe or recurrent gastrointestinal bleeding and to identify which patient populations would derive the greatest benefit.

## Conclusions

This study supports the hypothesis that bevacizumab, through targeted VEGF-A inhibition and suppression of pathological angiogenesis, may serve as an effective systemic salvage therapy for refractory gastrointestinal bleeding in HHT. The clinical response observed following induction therapy and the subsequent relapse upon treatment interruption underscores the disease-modifying potential of anti-VEGF therapy in this population. Unlike conventional endoscopic and pharmacologic strategies that address the downstream consequences of vascular malformations, bevacizumab targets the fundamental angiogenic dysregulation that drives HHT pathology. These findings have broader implications for the management of non-HHT gastrointestinal angiodysplasia, where aberrant VEGF signaling similarly contributes to pathological vessel formation and bleeding. Larger prospective randomized studies are urgently needed to establish evidence-based treatment protocols, including optimal dosing regimens, maintenance schedules, patient selection criteria, and long-term safety monitoring. Until such data are available, bevacizumab should be considered as a viable salvage option in carefully selected HHT patients with refractory GI bleeding who have failed conventional multimodal therapy.
